# Phosphatase-Dependent Regulation of Epithelial Mitogen-Activated Protein Kinase Responses to Toxin-Induced Membrane Pores

**DOI:** 10.1371/journal.pone.0008076

**Published:** 2009-11-30

**Authors:** Jorge L. Aguilar, Ritwij Kulkarni, Tara M. Randis, Sandeep Soman, Alexander Kikuchi, Yuxin Yin, Adam J. Ratner

**Affiliations:** 1 Department of Pediatrics, Columbia University, New York, New York, United States of America; 2 Department of Microbiology & Immunology, Columbia University, New York, New York, United States of America; 3 Department of Radiation Oncology, Columbia University, New York, New York, United States of America; University of Hyderabad, India

## Abstract

Diverse bacterial species produce pore-forming toxins (PFT) that can puncture eukaryotic cell membranes. Host cells respond to sublytic concentrations of PFT through conserved intracellular signaling pathways, including activation of mitogen-activated protein kinases (MAPK), which are critical to cell survival. Here we demonstrate that in respiratory epithelial cells p38 and JNK MAPK were phosphorylated within 30 min of exposure to pneumolysin, the PFT from *Streptococcus pneumoniae*. This activation was tightly regulated, and dephosphorylation of both MAPK occurred within 60 min following exposure. Pretreatment of epithelial cells with inhibitors of cellular phosphatases, including sodium orthovanadate, calyculin A, and okadaic acid, prolonged and intensified MAPK activation. Specific inhibition of MAPK phosphatase-1 did not affect the kinetics of MAPK activation in PFT-exposed epithelial cells, but siRNA-mediated knockdown of serine/threonine phosphatases PP1 and PP2A were potent inhibitors of MAPK dephosphorylation. These results indicate an important role for PP1 and PP2A in termination of epithelial responses to PFT and only a minor contribution of dual-specificity phosphatases, such as MAPK phosphatase-1, which are the major regulators of MAPK signals in other cell types. Epithelial regulation of MAPK signaling in response to membrane disruption involves distinct pathways and may require different strategies for therapeutic interventions.

## Introduction

Epithelial cells lining mucosal sites are in constant contact with microbial products. These cells are normally immunologically quiescent but have the capacity to respond rapidly to microbial threats by activating innate immune signaling. Some bacterial species produce protein toxins that may disrupt eukaryotic membrane integrity. These pore-forming toxins (PFT) are essential to virulence for many pathogens, and their prompt detection may be of benefit to the host. PFT are typically released as soluble monomers and bind to eukaryotic cell membranes, where they homo-oligomerize to form ring-shaped structures followed by functional pores [1]. In sufficient concentrations, PFT may lead to host cell cytolysis. We have previously demonstrated that epithelial cells detect the osmotic stress associated with sublytic concentrations of PFT and initiate immune responses through phosphorylation of p38 mitogen-activated protein kinase (MAPK) [2], [3]. MAPK signaling is a conserved response to a variety of cell stresses and is essential for survival of toxin-mediated membrane disruption [4], [5]. While PFT-induced osmotic stress has been linked to activation of MAPK signaling, the mechanisms involved in termination of this response are less clear. Proper regulation of MAPK activation is important in order to prevent excessive inflammatory responses that may lead to damage of host tissues.

The archetypal deactivator of MAPKs is MAP kinase phosphatase 1 (MKP1, also known as dual specificity phosphatase (DUSP)-1) [6]. Knockout of the *mkp1* gene is associated with prolonged MAPK activation and an increased likelihood of endotoxic shock after bacterial challenge [7], [8], [9], [10]. Upregulation of MKP1 expression is thought to be a major regulator of MAPK signaling and a crucial component of the termination of proinflammatory signaling. Here we show a novel, MKP1-independent mechanism for the regulation of the MAPK response to bacterial PFT. Using an important respiratory pathogen, *Streptococcus pneumoniae*, and its cognate PFT, pneumolysin (Ply), our studies demonstrate that epithelial termination of PFT-induced MAPK signals involves protein phosphatases 1 and 2A (PP1 and PP2A), but not MKP1. These findings indicate that epithelial cells may utilize signaling pathways for termination of immune responses that are distinct from those used in other cell types, especially professional immune cells, with implications for understanding homeostasis at mucosal surfaces.

## Results

### Pore Formation by *S. pneumoniae* Induces a Tightly Regulated MAPK Response in Respiratory Epithelial Cells

**Figure 1:**
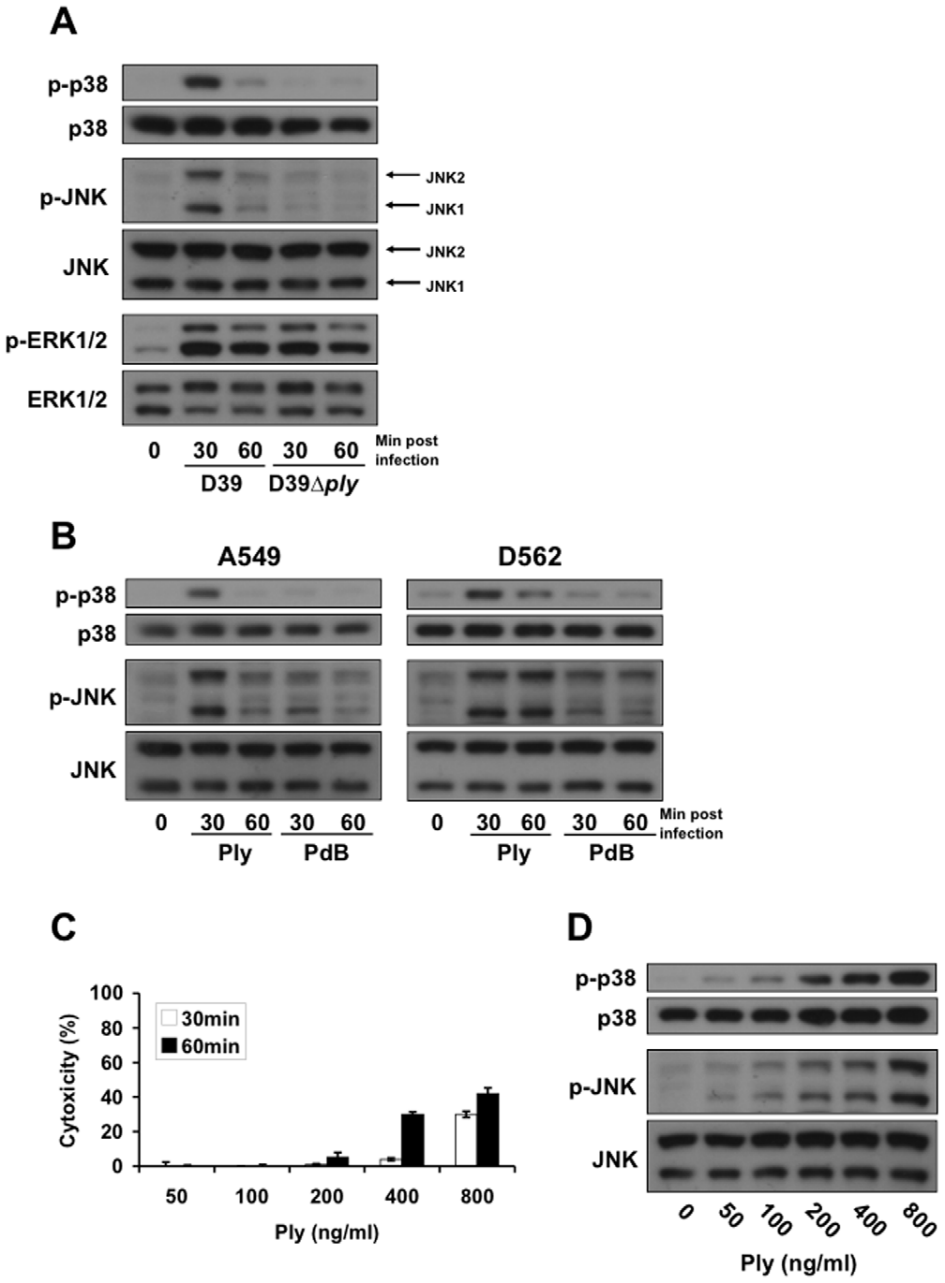
Subcytolytic pore-formation *by S. pneumoniae* leads to temporary activation of epithelial MAP kinases. (A) Confluent monolayers of A549 cells were stimulated for the indicated times with 4×10^4^ cfu/ml sonicated *S. pneumoniae* D39 or its isogenic Ply-deficient mutant, D39*ply*. Cells were subsequently lysed, and total and phosphorylated MAPK detected by western blot. Stimulation with D39, but not D39*ply*, leads to temporary phosphorylation of p38 and JNK MAPKs. (B) Stimulation of A549 and D562 respiratory epithelial cells for the indicated times with 200 ng/ml of purified Ply toxin but not its toxoid, PdB, induces MAPK activation. (C) A549 Cell lysis was measured by LDH assay of cell supernatants with triplicate samples. (D) Dose-dependent MAPK phosphorylation in A549 cells treated with indicated concentrations of Ply for 30 min.

We have previously shown that epithelial cells detect the formation of membrane pores by bacterial PFTs, including Ply from *S. pneumoniae*, via an intracellular signaling pathway involving the phosphorylation of MAP kinases [3]. To investigate the regulatory timeline of this detection mechanism, A549 respiratory epithelial cells were treated with *S. pneumoniae* D39 or its isogenic Ply-deficient mutant D39*ply* for up to 60 min and the time-dependent phosphorylation of MAP kinases evaluated by immunoblot analysis. Treatment of A549 cells with D39, but not with an equivalent number of D39*ply*, led to the phosphorylation of p38 and JNK kinases (Fig. 1A). Additionally, both kinases underwent dephosphorylation within 1 hour after activation, suggesting that this signaling pathway is subject to timely negative regulation. To determine whether regulation of the MAPK response is due to the effect of pore-formation, we performed a similar experiment using purified Ply and the Ply toxoid, PdB, which inserts and oligomerizes in membranes but does not form functional pores [11]. Treatment of A549 and D562 respiratory epithelial cells with purified Ply, but not the PdB toxoid, led to similar p38 and JNK MAPK activation timelines (Fig. 1B), indicating that the observed responses are not specific to a single cell line. Treatment with D39, D39*ply*, Ply, and PdB all induced activation of ERK1/2 kinases (Fig. 1A–B), but this property may not be due to pore formation, as there was a similar response to the Ply-deficient mutant and the PdB toxoid. The ng/ml concentrations of Ply toxin used in this report did not affect cell viability as determined by measurement of cellular LDH release (Fig. 1C). Increasing concentrations of toxin led to a dose-dependent increase in MAPK phosphorylation (Fig. 1D).

### Termination of the Epithelial MAPK Response to Pore Formation Is Mediated by Phosphatase Activity

**Figure 2:**
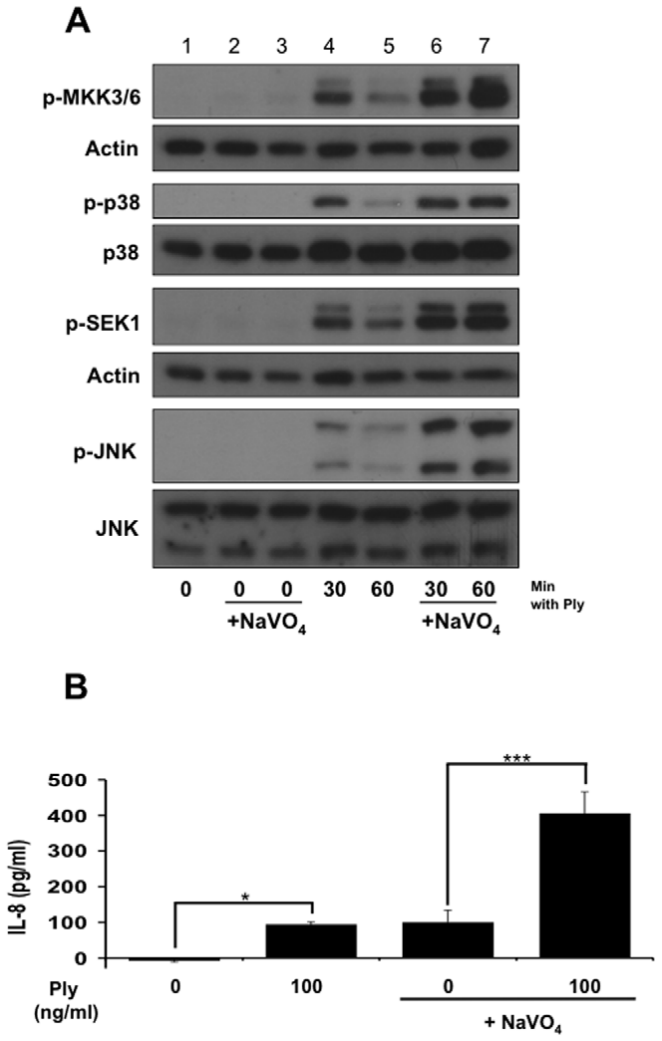
Inactivation of the epithelial innate immune response to pore-formation is mediated by phosphatase activity. (A) Confluent monolayers of A549 cells were stimulated for the indicated times with 200 ng/ml of purified Ply after pretreatment with either 100 µM sodium orthovanadate (lanes 6, 7) or vehicle control (lanes 4, 5) for 30 min. As corresponding negative controls, cells were left untreated (lane 1) or treated with sodium orthovanadate alone for 60 and 90 min (lanes 2 and 3, respectively). Sodium orthovanadate leads to enhanced Ply-induced phosphorylation of p38, JNK, and their respective upstream kinases, MKK3/6 and SEK1. (B) Pretreatment of A549s with 100 µM sodium orthovanadate (30 min) leads to significantly higher production of Ply-induced IL-8 than vehicle control (*  =  ≤0.05, ***  =  ≤0.001 by ANOVA with Tukey post-test).

MAPK phosphorylation is a reversible process that is controlled tightly by the activity of protein phosphatases [12]. To explore a functional role for phosphatases in the epithelial MAPK response to Ply, we used sodium orthovanadate (NaVO_4_), a competitive inhibitor of protein phosphatases. Compared with control cells, those pretreated with NaVO_4_ exhibited more intense and longer lasting MAPK phosphorylation after Ply stimulus (Fig. 2A), indicating that phosphatase activity mediates negative regulation of the Ply induced MAPK response. The effect of sodium orthovanadate appeared to involve upstream kinases SEK1 and MKK3/6 as well, indicating that the targeted phosphatases may act at multiple levels of this signaling pathway.

### Phosphatase Inhibition Enhances Epithelial Cytokine Responses

Because of the relationship between MAPK activation and stabilization of cytokine mRNAs, we investigated whether phosphatase inhibition would alter toxin-induced epithelial IL-8 production. Treatment of cells with sodium orthovanadate primed epithelial responses to pneumolysin, leading to a significant increase in IL-8 production as measured by ELISA (Fig. 2B).

### MKP1 Does Not Regulate the Epithelial MAPK Response to Pore Formation

**Figure 3:**
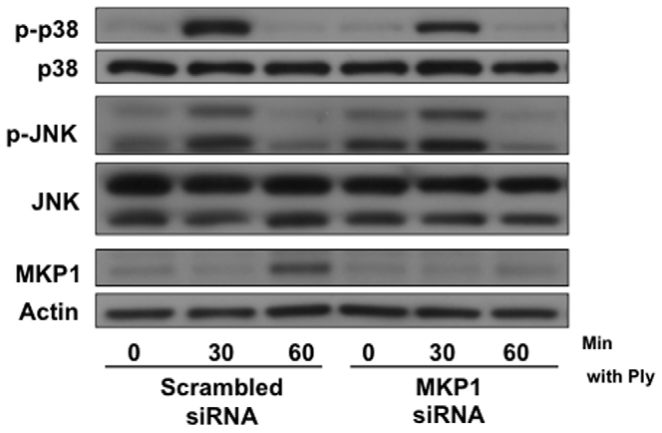
Regulation of the MAPK response to pore-formation is MKP1 independent. (A) A549 cells were transfected with 2.5 µg of MKP1 or scrambled siRNA per 1×10^6^ cells and stimulated 24 hrs post-transfection with 200 ng/ml of purified Ply for the indicated times. Transfection with MKP1 siRNA inhibited MKP1 expression but did not have an effect on Ply-induced MAPK phosphorylation.

Proven to directly dephosphorylate p38 and JNK kinases in numerous cellular signaling mechanisms [6], MKP1 was investigated first in order to identify a negative regulator of the MAPK response to pore-formation. Treatment of A549s with Ply induced expression of MKP1 that corresponded temporally with deactivation of p38 and JNK. siRNA-mediated knockdown of cellular MKP1 expression did not affect the magnitude or kinetics of MAPK activation (Fig. 3).

### Pharmacological Inhibition of Serine/Threonine Phosphatases Enhances the Ply Induced MAPK Response

**Figure 4:**
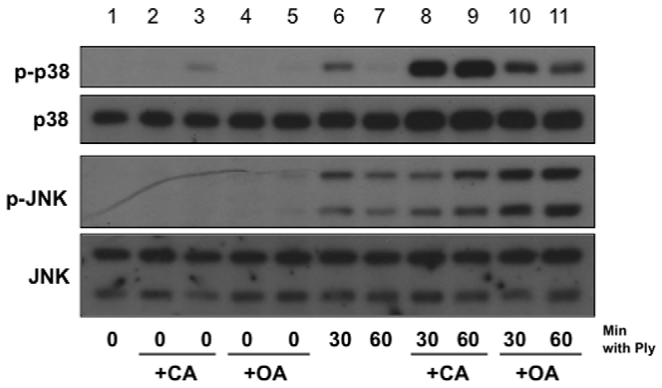
Inhibition of serine/threonine phosphatases leads to increased Ply-induced epithelial MAPK phosphorylation. Confluent monolayers of A549 cells were stimulated for the indicated times with 100 ng/ml of purified Ply after pretreatment with either 5 nM calyculin A for 30 mins (lanes 8, 9), 250 nM okadaic acid for 60 mins (lanes 10, 11), or vehicle control (lanes 6, 7) for 30 mins. As corresponding negative controls, cells were left untreated (lane 1) or treated with calyculin A alone for 30 and 60 mins (lanes 2 and 3, respectively) or okadaic acid alone for 60 and 90 mins (lanes 4 and 5, respectively). In both cases, pretreatment with serine/threonine phosphatase inhibitors leads to prolonged and enhanced p38 and JNK phosphorylation in response to Ply.

Several lines of evidence suggest that serine/threonine phosphatases, including PP1 and PP2A, deactivate MAPKs during cellular stress responses [13], [14], [15], [16], [17], [18]. To investigate whether these phosphatases serve a regulatory role in the MAPK response to pore formation, we used pharmacological inhibitors, calyculin A and okadaic acid. A549 cells pretreated with either calyculin A or okadaic acid exhibited greater Ply-induced MAPK phosphorylation than untreated cells (Fig. 4). Both inhibitors prolonged and enhanced p38 and JNK phosphorylation by Ply, but calyculin A appeared to exhibit a greater effect on p38, while okadaic acid had a greater effect on JNK. Taken together, these data suggest that serine/threonine phosphatases may serve important, not entirely redundant functions in the MAPK response to Ply.

### PP1 and PP2A Act as Negative Regulators of Ply iInduced MAPK Activation

**Figure 5:**
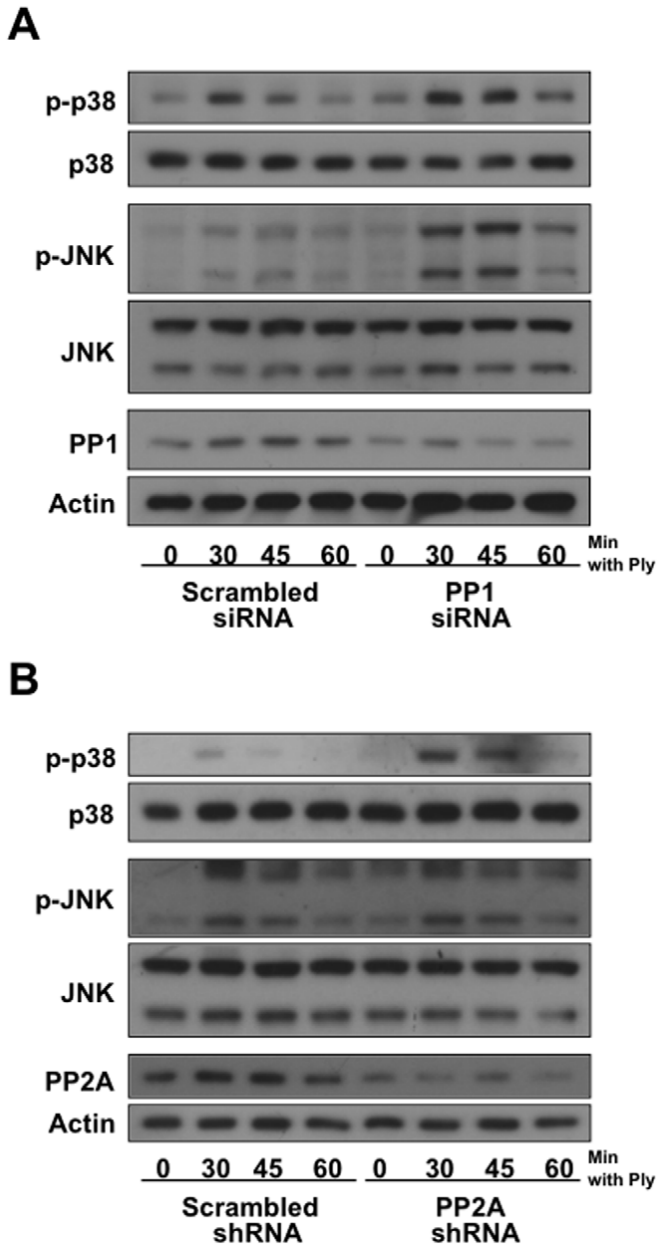
PP1 and PP2A mediate inactivation of the epithelial MAPK response to pore-formation. (A)A549 cells were transfected with 2 µg PP1 or scrambled siRNA per 1×10^6^ cells and stimulated 54 hrs post-transfection with 100 ng/ml of purified Ply. Transfection with PP1 siRNA leads to reduced PP1 expression and a corresponding increase in Ply-induced p38 and JNK phosphorylation. (B) A549 cells were transfected with 6 µg PP2Aα/β or control shRNA per 1×10^6^ cells and stimulated 60 hrs post-transfection with 100 ng/ml of purified Ply. Transfection with PP2Aα/β shRNA leads to reduced PP2A expression and a moderate increase in p38 phosphorylation.

To ascertain whether PP1 could regulate MAPK activation by Ply, siRNA targeting PP1 was transfected into A549 cells and MAPK phosphorylation assayed. PP1 expression was reduced in cells treated with PP1 siRNA, and Ply-induced phosphorylation of p38 and JNK was concomitantly increased and prolonged (Fig. 5A), though the effect appeared to be more pronounced on JNK. Knockdown of PP2A was achieved using plasmid-mediated shRNA specifically targeting the different isoforms of subunit A [19], an essential component of the heterotrimer phosphatase [20], [21]. Reduced expression of PP2A led to prolonged p38 but not JNK phosphorylation after treatment with Ply (Fig. 5B). In both cases, RNAi-mediated knockdown of the cellular phosphatases was incomplete, but effects on MAPK activation states were observed. Collectively, these data indicate an involvement of serine/threonine phosphatases PP1 and PP2A in negative regulation of the epithelial MAPK response to pore-formation.

## Discussion

Epithelial cells lining mucosal surfaces may detect the composition and density of the colonizing microbial flora and subsequently initiate local innate immune responses. The professional immune cells that are recruited may then reduce the population size of potentially threatening microbes. However, if the magnitude or duration of inflammation is inappropriately large, the outcome may be detrimental to the host. Excessive inflammation can damage local host tissues and render them susceptible to chronic infection, as is the case in inflammatory bowel disease and Crohn's disease. Timely negative regulation of innate immune responses thus serves a critical function in host homeostasis and can influence the outcome of syndromes such as septic shock [8], [9].

When epithelial cells are exposed to subcytolytic concentrations of bacterial PFT, a variety of host signaling pathways become engaged. In some cases, receptor-based recognition of specific toxin structures appears to be important in the initiation of host responses [22]. However, conserved cellular activities in response to membrane disruption (termed “cellular non-immune defenses” by Aroian and van der Goot [23]) likely constitute the initial responses to PFT. Sensation of sublethal osmotic stress induced by PFT leads to epithelial MAPK phosphorylation. Once activated, these MAPKs continue a signaling cascade leading to the production of pro-inflammatory cytokines [3]. Sublethal concentrations of PFT modulate a variety of other host cell signaling pathways. Wiles et al. showed potent inhibition of host Akt and protein kinase B signaling in response to HlyA, a PFT from *E. coli*, as well as unrelated PFT [24]. This mechanism, which impacts host cell survival, was dependent on the activation of phosphatases in target cells. Our observations in these studies are in agreement with their findings. The ability to briskly activate MAPK signaling in response to bacterial PFT is a critical determinant of target cell survival [4], and downstream signaling, including activation of the unfolded protein response, has been shown to mediate such survival [5]. Immunoglobulins targeting bacterial cytolysins may alter cellular survival as well [25]. Other, non-MAPK signaling including lipid metabolic pathways, caspase activation, and membrane repair play major roles in the overall host cell response to bacterial PFT [26], [27], [28].

While it has become increasingly understood that MAPK signaling is an integral part of cellular non-immune defenses to bacterial PFT, the mechanisms involved in temporal regulation of these pathways are less clear. Regulatory pathways may be complex and cell-type specific [29]. In other systems, dual specificity phosphatases including MKP1 are critical for negative feedback control of MAPKs [12]. We found that MKP1 expression was induced in epithelial cells treated with Ply (Fig. 3), but our results indicated that it does not play a major role in termination of MAPK signals in these cells. In contrast, our data are most consistent with regulation of PFT-induced MAPK signals by PP1 and PP2A serine/threonine phosphatases.

Both PP1 and PP2A have been shown to regulate MAPK signaling under specific circumstances [13], [14], [15], [16], [17], [18], [30]. However, their roles are generally thought to be less important than those of the dual-specificity phosphatases such as MKP1. We have demonstrated overlapping but not completely redundant roles for these phosphatases in termination of MAPK signaling following toxin-induced membrane disruption. Given the important role of MAPK signaling in mechanical lung injury, such regulation may have implications for lung homeostasis in non-infectious disease states as well [31]. These findings are important for further elucidation of cellular non-immune defense pathways as well as for charting and, ultimately, modulating host defense pathways during infections such as pneumococcal pneumonia, in which Ply is important and the balance between inflammatory and anti-inflammatory signaling may be a critical determinant of outcome [32].

## Methods

### Bacterial Strains and Products

*S. pneumoniae* strains D39 [33] and its Ply-deficient derivative D39*ply* [34], kindly provided by D. Briles (University of Alabama), were grown as described [35], [36]. Recombinant Ply was produced as described [2]. PdB (Ply-W433F) was made by site directed mutagenesis of the pET29a/Ply plasmid using a Quik-change II XL kit (Stratagene) and primers PLYW433F-F (5′-TAGAGAGTGTACCGGGCTTGCCTTTGAATGGTGGCGTACGGTTTAT-3′) and PLYW433F-R (5′-ATAAACCGTACGCCACCATTCAAAGGCAAGCCCGGTACACTCTCTA-3′).

### Epithelial Cell Lines and Culture Conditions

A549 (CCL-185) and D562 (CCL-138) cells were obtained from ATCC and grown in modified Eagle's medium (MEM) (Invitrogen) supplemented with 1 mM sodium pyruvate, 10% fetal bovine serum (HyClone), and 10 µg/ml of ciprofloxacin. Cells were weaned from serum and antibiotics for 24 hrs prior to experiments.

### Phosphatase Inhibitors

General phosphatase activity inhibitor, sodium orthovanadate, was purchased from Sigma-Aldrich (St. Louis, MO). Serine/threonine phosphatase inhibitors, calyculin A and okadaic acid, were purchased from Cell Signaling (Danvers, MA) and Calbiochem (San Diego, CA), respectively.

### Plasmids and Transfections

#### Transfections

To maximize transfection efficiencies, A549 cells were transfected using the Amaxa Nucleofector II system (Lonza, Germany) as described by their cell type-specific protocols.

### siRNA and shRNA

MKP1 siRNA was from Dharmacon (Lafayette, CO). PP1 and scrambled siRNAs were from Santa Cruz Biotechnology (Santa Cruz, CA), and PP2Aα/β and GFP control shRNA were constructed by William Hahn and obtained from Addgene (Cambridge, MA) (Addgene plasmids 10676, 15249, and 15250) [19].

### Stimulation of Epithelial Cells, LDH Assay, Western Blotting, and ELISA

A549 and D562 respiratory epithelial cells were grown to confluence in 12-well plates and were weaned from serum and antibiotics prior to treatment. Sonicated bacteria were prepared as previously described [3] and 4×10^4^ colony-forming unit equivalents/ml of *S. pneumoniae* were added to the epithelial monolayer. Where indicated, phosphatase inhibitor pretreatments were prepared by dilution with MEM and remained present during exposure to Ply. All pretreatments, bacteria and bacterial toxins were incubated with epithelial cells at 37°C and 5% CO_2_ for the indicated durations. To confirm given treatments as subcytolytic, supernatants were collected and lactate dehydrogenase release assessed using a commercial kit (Cytotoxicity Detection Kit Plus; Roche Applied Science). After washing with sterile PBS, cells were lysed on ice in RIPA lysis buffer with protease and phosphatase inhibitors. Aliquots with equal amounts of protein were loaded and separated on a 4–12% bis-tris gel (NuPAGE; Invitrogen). Proteins were transferred to polyvinylidene difluoride membranes (Immobilon P; Millipore) and probed using specific phosphatase and phospho-MAPK antibodies as indicated. To control for loading amounts, blots were subsequently stripped and reprobed to detect actin or total MAPK. Western experiments were performed a minimum of three times, and a representative experiment is presented. Antibodies against phospho-p38, total p38, phospho-JNK, total JNK, phospho-ERK, total ERK, phospho-SEK1, and phospho-MKK3/6, and the catatytic subunit of PP2A were purchased from Cell Signaling (Danvers, MA). Antibodies against MKP1, PP1 and actin were from Santa Cruz Biotechnologies (Santa Cruz, CA). For experiments involving Ply-induced interleukin (IL)-8 production, cells were treated with 200 ng/ml Ply for 1 hr, the Ply-containing medium was removed, the cells were washed three times and then incubated in fresh medium overnight. The concentration of IL-8 in cell supernatants was determined by ELISA (BD OptEIA) according to the manufacturer's instructions.
